# The compact genome of *Caenorhabditis niphades* n. sp., isolated from a wood-boring weevil, *Niphades variegatus*

**DOI:** 10.1186/s12864-022-09011-8

**Published:** 2022-11-22

**Authors:** Simo Sun, Natsumi Kanzaki, Mehmet Dayi, Yasunobu Maeda, Akemi Yoshida, Ryusei Tanaka, Taisei Kikuchi

**Affiliations:** 1 Department of Integrated Biosciences, Graduate School of Frontier Sciences, The University of Tokyo, Chiba, 277-8562 Japan; 2 Department of Infectious Diseases, Faculty of Medicine, University of Miyazaki, 5200 Kihara, Miyazaki, 889-1692 Japan; 3 Kansai Research Center, Forestry and Forest Products Research Institute, 68 Nagaikyutaroh, Momoyama, Fushimi, Kyoto, 612-0855 Japan; 4 Forestry Vocational School, Duzce University, 81620 Duzce, Türkiye; 5 Genomics and Bioenvironmental Science, Frontier Science Research Center, University of Miyazaki, Miyazaki, 889-1692 Japan

**Keywords:** *C. elegans*, Chromosomal level assembly, Small-genome size, Insect-associated

## Abstract

**Background:**

The first metazoan genome sequenced, that of *Caenorhabditis elegans*, has motivated animal genome evolution studies. To date > 50 species from the genus *Caenorhabditis* have been sequenced, allowing research on genome variation.

**Results:**

In the present study, we describe a new gonochoristic species, *Caenorhabditis niphades* n. sp., previously referred as *C.* sp. 36, isolated from adult weevils (*Niphades variegatus*), with whom they appear to be tightly associated during its life cycle. Along with a species description, we sequenced the genome of *C. niphades* n. sp. and produced a chromosome-level assembly. A genome comparison highlighted that *C. niphades* n. sp. has the smallest genome (59 Mbp) so far sequenced in the Elegans supergroup, despite being closely related to a species with an exceptionally large genome, *C. japonica*.

**Conclusions:**

The compact genome of *C. niphades* n. sp. can serve as a key resource for comparative evolutionary studies of genome and gene number expansions in *Caenorhabditis* species*.*

**Supplementary Information:**

The online version contains supplementary material available at 10.1186/s12864-022-09011-8.

## Significance statement

We describe a new species, *Caenorhabditis niphades* n. sp., characterized by the smallest genome size with chromosome-level genome assembly within the Elegans supergroup described so far. Considering its close phylogenetic position with *Caenorhabditis japonica*, which conversely possesses one of the largest genomes in the group, *Caenorhabditis niphades* n. sp. can serve as a model for the analysis of genome size evolution in nematodes.

## Background

The nematode *Caenorhabditis elegans* is an outstanding model organism used in several modern biology fields [1–3]. However, in contrast to its well-studied genetics, development, neurobiology, and other molecular mechanisms, little is known about their life history and ecology. Nevertheless, recent sampling efforts have led to the identification of numerous wild *C. elegans* strains [4] and relative species, [5–7] including *C. inopinata,* the sister species of *C. elegans* [8], allowing the study of their genetic diversity. Species in the genus *Caenorhabditis* are morphologically similar to each other but very diverse ecologically [9]. Among the approximately 65 *Caenorhabditis* species identified so far, most were isolated from organic-substance-rich environments such as decayed stems and rotten plants, showing a “free-living” lifestyle with only weak (phoretic) association to other invertebrate animals; however, some species are known to tightly associate with other invertebrates in their lifecycle. For example, *C. japonica* has an obligate association with a shield bug thorough their whole lifecycle [10] and *C. inopinata* requires a specific wasp as a vector to continue their lifecycle [8]. Moreover, *C. bovis* was isolated from Zebu’s (*Bos indicus*) inflamed ears, where it is associated with bovine otitis [11]. These *Caenorhabditis* species/isolate collections are not only a useful resource for comparative studies aiming to understand nematode diversity and evolution but also to provide an evolutionary context for the biological phenomena observed in *C. elegans* laboratory strains. Genome sequences of those species further help our understanding of *C. elegans* biology and evolutionary forces that have shaped its genome. Currently, draft genomes are available for > 50 *Caenorhabditis* species and approximately seven species have high-quality chromosomal level assemblies [8, 11–16]. Genome comparisons revealed genome diversity in the genus, including with respect to gene repertories and genome size. For instance, a recent genome comparison of 24 *Caenorhabditis* species revealed genome size variation from 65 Mb (*C. afra* and *C. sulstoni*) to 140 Mb (*C. doughertyi*) [7].

Here, we describe a new gonochoristic species, *Caenorhabditis niphades* n. sp., previously referred as *C.* sp. 36, which was isolated from adult weevils (*Niphades variegatus*), with whom they appear to be tightly associated in their lifecycle. Along with a species description, we not only sequenced *C. niphades* n. sp.’s genome but also produced a chromosome-level assembly. *C. niphades* n. sp. was shown to have a 59 Mb genome, the smallest in the Elegans supergroup.

## Results

We isolated nematodes from *Niphades variegatus,* a wood-boring weevil that inhabits newly dead logs of Pinaceae trees, as described in Materials and Methods. Several nematode cultures were established from single females and subjected to morphological observation and ribosomal DNA sequencing. Among them, we identified a new nematode species *Caenorhabditis niphades* n. sp. (formerly known as *C.* sp. 36). Nematodes showing identical ribosomal DNA sequences were isolated multiple times from *N. variegatus* collected in distinct locations of Japan (Esashi, Sapporo, Hachioji, Ueda and Kyoto) (Fig. S1), whereas *C. niphades* n. sp. has not been found from other insects co-habiting with *N. variegatus* (e.g., bark and longhorn beetles and other weevil species). Accordingly, the new species *C. niphades* is considered to be widely distributed in Japan, and to use the weevil as primary carrier insect.

### Species description

A detailed taxonomic description is shown in the Supplementary information 1. Only the relevant diagnostic characteristics are described here and later in the Result section. The general typological characters of *C. niphades* n. sp. are conserved as in those of other *Caenorhabditis* species of the Elegans supergroup (Figs. S2-S5, Table S1) [5, 6, 17–19]. Within Elegans supergroup, *C. niphades* n. sp., however, can be diagnosed by its relatively wide stoma, i.e., width-length ratios are ca. 4.2 and 3.8 for males and females, respectively, and its composition, i.e., the ratio of chailo, gymno and stegostom is 1: 1: 2. This combination has not been reported in the other species in Elegans supergroup [5, 6, 8, 17].

### Phylogeny

A preliminary phylogenetic analysis based on the comparison of 18S and 28S ribosomal RNA gene sequences between *C. niphades* n. sp. and other 28 *Caenorhabditis* species confirmed that *C. niphades* n. sp. is a distinct species related to other Elegans supergroup species including *C. elegans* (Fig. S1). In a previous extensive phylogenetic analysis, the genus *Caenorhabditis* has been divided into subgroups including the Elegans supergroup, comprising Elegans and Japonica groups, and the Drosophilae supergroup [5, 7, 20]. However, the Elegans and Japonica groups are not well separated in the ribosomal RNA-based tree.

**Fig. 1:**
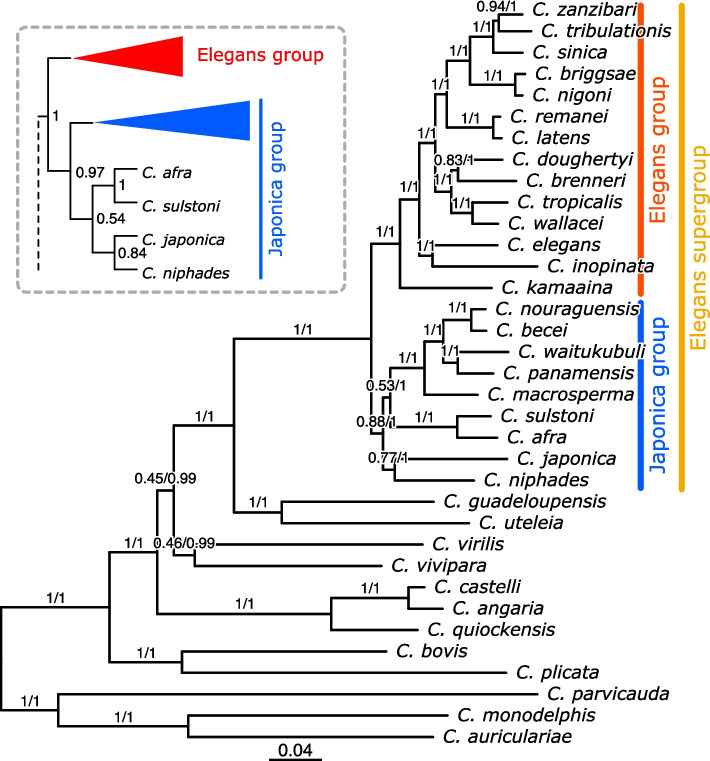
Phylogeny of *Caenorhabditis niphades* n. sp. A total of 308 single-copy orthologues from 35 *Caenorhabditis* species were used to generate trees using Maximum-likelihood (ML) and Bayesian inference (BI) approach with a concatenated alignment. The ML and BI trees shown as a main figure showed an identical topology to each other. Supertree (ST) approach was also employed to infer species tree, using independent gene trees as input. The ST tree showed the same topology as the ML/BI except for the relationship within the Japonica group species which was shown in the inset. Support values were shown on each branch of the trees (ML/BI)

We then performed a genome-based phylogenetic analysis to more precisely investigate the phylogenetic relationships of *C. niphades* n. sp. with other species of the genus. A total of 308 single-copy genes from *C. niphades* n. sp. (please see below) and 35 other *Caenorhabditis* species were obtained and used to generate phylogenetic trees using Bayesian inference (BI), Maximum-likelihood (ML) and Supertree (ST) approaches (Fig. 1). The resulting BI and ML trees showed the same topology to each other. In these trees, *C. niphades* n. sp. belonged to the Japonica group, being clustered with *C. japonica,* even though the branches of the two species were relatively long. The Supertree (ST) approach, using independent gene trees, confirmed the close relationship of *C. niphades* n. sp. and *C. japonica* despite a slightly different topology for the other species within Japonica group. These disagreements possibly indicate complex relationships among these species as described by Stevens et al. [11].

### *Genome characteristics* of *C. niphades* n. sp.

**Fig. 2:**
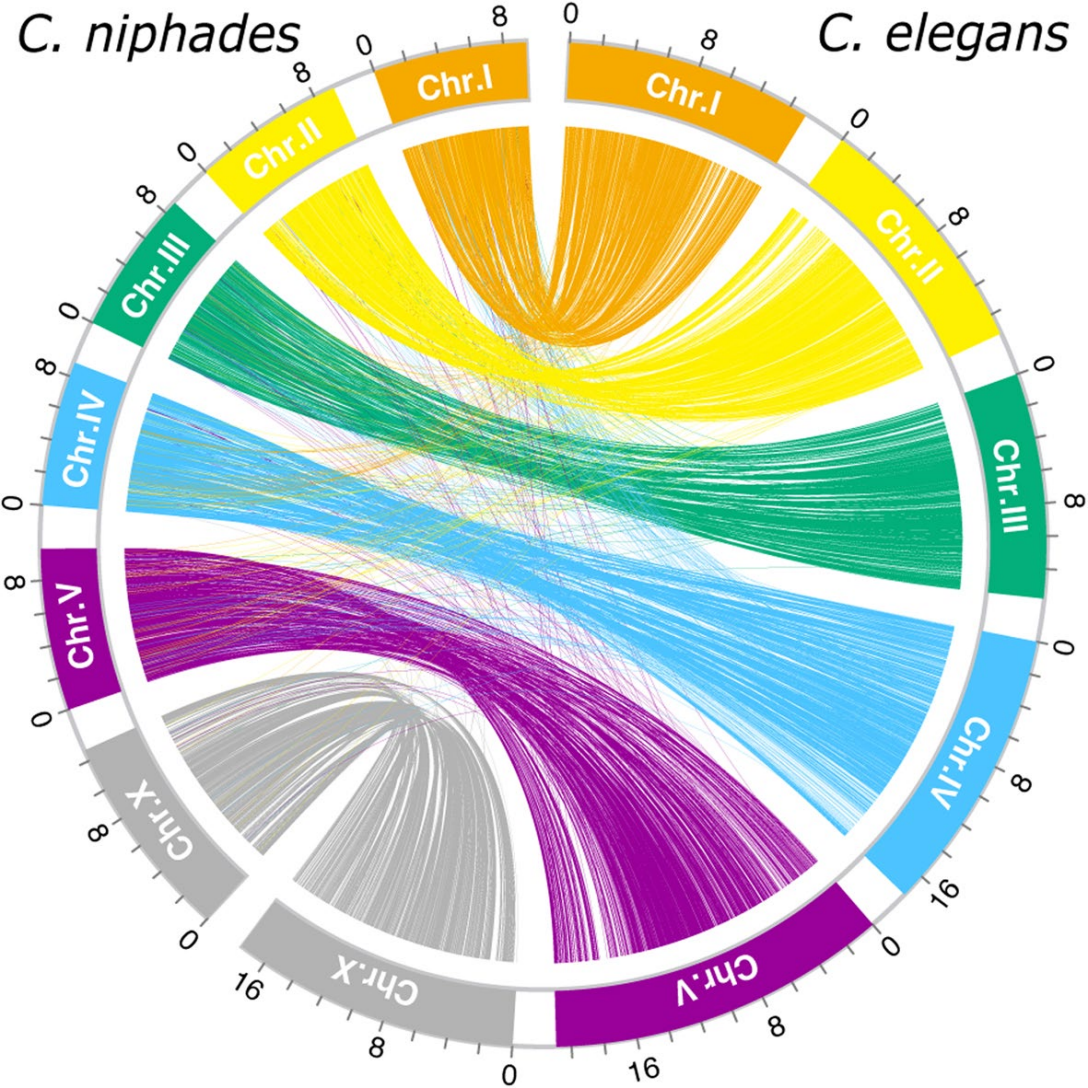
A map connecting syntenic regions between the *C. niphades* n. sp. and *C. elegans* genomes. Syntenic regions were identified using 9105 single-copy orthologues between the two species

**Fig. 3:**
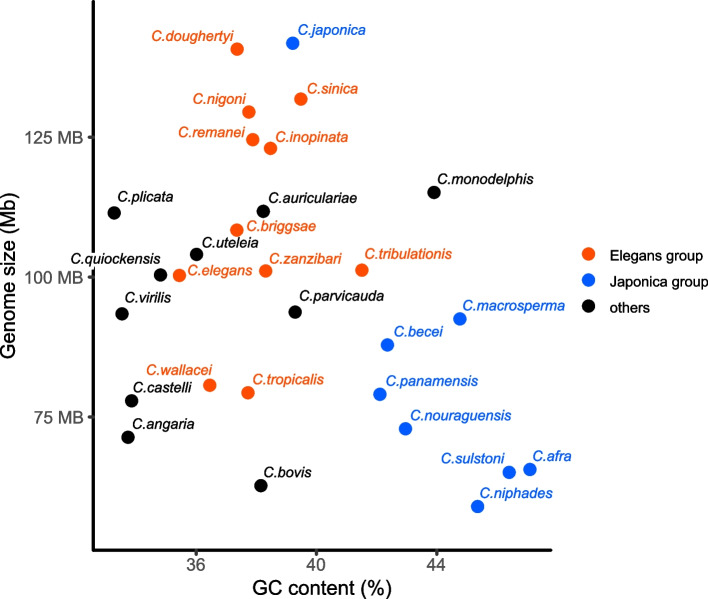
The correlation between GC content (%) and genome size (Mb) in *Caenorhabditis* species. Negative correlation was observed between G/C content and genome size in the Japonica group (R^2^ = 0.61, *p* = 0.01). The Japonica group (blue), Elegans group (red) and others (black), including the Drosophilae supergroup and basal species, are shown

We sequenced and assembled the genome of *C. niphades* n. sp. by a combination of Nanopore long reads, Illumina short reads, and Hi-C technology producing a highly-contiguous assembly. More than 99.9% of the assembly was assigned to six scaffolds (> 8 Mbp) with only four small contigs (< 10 kb) remaining unassigned (Table 1). The six scaffolds likely represent *C. niphades* n. sp.’s chromosomes as telomere signatures (TTAGGC)_n_ observed for four of them (Fig. S6). The Hi-C contact map showed six clear clusters with strong contact with other scaffolds in the center, except for one big chromosome (Fig. S7), which may due to similar mechanisms as those described in other *Caenorhabditis* species having perinuclear anchoring [14]. We designated the six scaffolds as ChrI–ChrV and ChrX based on their synteny with *C. elegans’* chromosomes (Fig. 2). The 59.0 Mb assembly has high completeness (97.4% as assessed by BUSCO; Table 1), but is 40% smaller than the *C. elegans* genome, representing the smallest haploid genome reported in the Elegans supergroup. We found two other small-genome species in the Japonica group (*C. afra* and *C. sulstoni*), whereas *C. japonica* shows the largest genome size (141.2 ~ 166.3 Mb) in the Japonica group. A comparison in the proportion of G/C to A/T bases (GC content) of the genomes of species in the Japonica group showed a clear negative correlation between genome size and GC content, while the Elegans group and others did not show such a trend (Fig. 3). The three species of the Japonica group, *C. niphades* n. sp., *C. afra,* and *C. sulstoni*, showed the highest GC content (45.4–47.1%) in the genus and the smallest genome sizes (59.0, 65.6, and 65.1 Mb, respectively; Fig. 3). *C. bovis*, belonging to the basal genus group possesses a small-genome size (62.7 Mb) but with ~ 38% GC content.

**Table 1:** Genome statistics of *C. niphades* n. sp. and other selected *Caenorhabditis* species

	***C. niphades*** (v5.5)	***C. elegans*** (WBPS14)	***C. bovis*** (v1.0)	***C. afra*** (JU1286_v1)	***C. sulstoni*** (JU2788_v1)	***C. japonica*** (WBPS16)	***C. japonica*** (v2.0)
Assembly size (Mb)	59.0	100.3	62.7	65.6	65.1	166.3	141.2
Number of scaffolds	6	6	35	1898	2044	662	39
Average (kb)	5900	14,327	1792	34.6	31.8	8.8	3840
Largest scaffold (kb)	12,682	20,924	10,858	1054	1009	1100	26,862
N50 (kb)	9392	17,493	7558	176	137	94	23,717
Gaps (kbp)	3	0	0	204	381	12,198	26
GC (%)	45.4	35.4	38.2	47.1	46.4	39.2	39.2
Num. coding genes	16,929	20,277	13,128	19,834	18,192	29,935	N/A
BUSCO genome completeness (%) single/duplicated	97.4/0.5	98.8/0.5	94.2/1.6	97.0/0.5	97.9/0.5	92.4/1.6	94.6/0.3

We predicted 16,929 protein-coding genes on the genome of *C. niphades* n. sp. Although this number is ~ 3000 smaller than that determined for *C. elegans*, most core genes were conserved in *C. niphades* n. sp. (Table 1, Fig. S8)*.* We generated orthologous groups (orthogroups) using 35 *Caenorhabditis* species and selected a core orthologue set (10,548 orthogroups) for the Elegans supergroup based on four well-annotated genomes (*C. elegans, C. inopinata, C. nigoni*, and *C. briggsae*). Accordingly, we found that 96.7% of the core orthologues were present in the *C. niphades* n. sp. genome whereas only 85.7% are conserved in *C. bovis*. The core orthologue set of *C. niphades* n. sp. included 8881 single-copy orthologues and 1323 multi-copy orthogroups consisting of 3799 genes. *C. elegans* has a similar number of single-copy core genes (8901) and multi-copy core orthogroups (1647) as *C. niphades* n. sp., but the gene number of multiple-copy core genes almost doubled (6273) that of *C. niphades* n. sp., implying that the gene number difference between *C. niphades* n. sp. and *C. elegans* is mainly due to repeated duplications of certain *C. elegans’* genes (Fig. S8). Taken together, the genome of *C. niphades* n. sp. appears to be a comprehensive and compact *Caenorhabditis* genome.

**Fig. 4:**
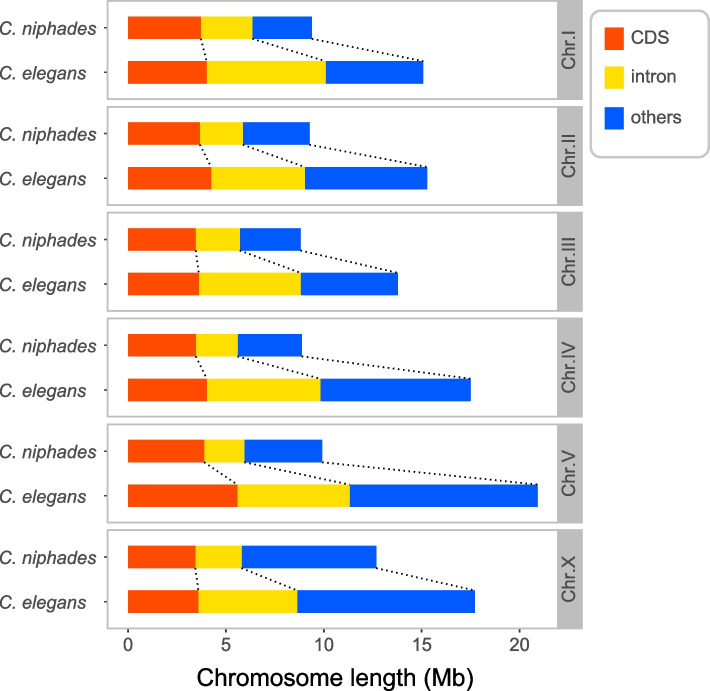
Chromosome size comparison between *C. niphades* n. sp. and *C. elegans*. The total length of CDS (blue), intronic (green), and intergenic (red) regions on each chromosome are shown in the bar plot.

*C. niphades* n. sp. and *C. elegans’* genomes have a 41.3 Mb size difference, largely due to differences in the intron span (19.0 Mb) and intergenic regions (18.9 Mb); only 3.4 Mb difference was due to the CDS span (Fig. 4). *C. niphades* n. sp.’s genome possesses 6.6 Mb transposable elements (TEs), accounting for 11.3% of the genome, whereas 17.4 Mb (17.4%) of TEs were detected in *C. elegans’* genomes (Table S2). Those TEs were mainly identified in introns and intergenic regions.

**Fig. 5:**
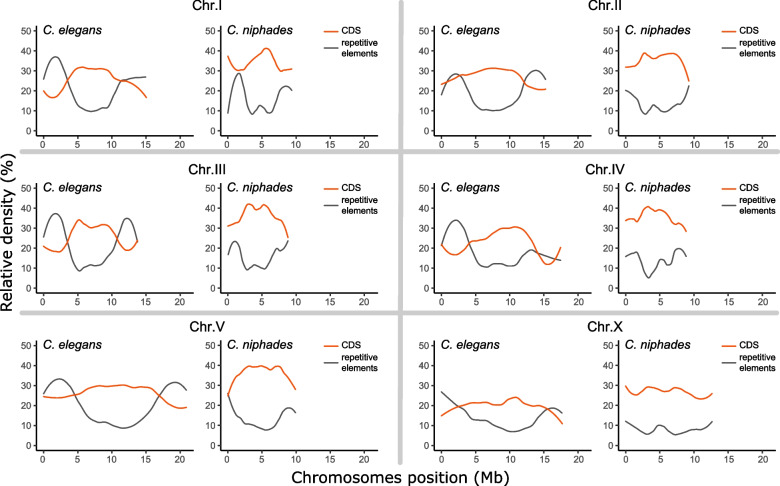
Distribution of repetitive elements and CDS in *C. niphades* n. sp. and *C. elegans*. Panels are separated by chromosomes. Smooth lines for proportions of repetitive elements and CDS contents in 100-kb sliding windows are shown in each plot in black and red, respectively

At chromosome-level, we found all six chromosomes of *C. niphades* n. sp. are smaller in length than those in *C. elegans* (Fig. 5). *C. elegan’s* chromosomes range from 13.8 Mb to 20.9 Mb, whereas those of *C. niphades* n. sp. except for ChrX (12.7 Mb) are < 10 Mb. The largest size difference was found in ChrV, where *C. niphades* n. sp.’s is only 47.4% the size of *C. elegans’*, followed by ChrIV (50.7%) and ChrII (60.7%). As a consequence of the small chromosomes, CDS densities of *C. niphades* n. sp. are higher than those of in *C. elegans* in all six chromosomes, where we observed higher CDS densities in the centre than in the arm of all chromosomes except ChrX of *C. niphades* n. sp. (Fig. 5). The repetitive elements show inverse patterns to CDS distribution (Fig. 5). This intracromosomal non-uniformity has also been reported in other *Caenorhabditis* species [11]. A comparison of gene number differences, CDS spans, intron spans, and intergenic region length determined that, in all chromosomes, gene number differences linearly increase with differences in CDS spans. ChrV showed the largest difference in gene number (1759 fewer genes in *C. niphades* n. sp.), followed by ChrII (585 genes) and ChrIV (583 genes). ChrX is the only exception where the gene number is bigger in *C. niphades* n. sp. (Table S3). The high gene number in *C. elegans’* ChrV can be partly explained by the massive expansion of GPCR gene families in *C. elegans* [8, 11], a signature not identified in *C. niphades* n. sp. (Table S3). Relationship between gene number differences and intergenic region spans, or intron spans varied in each chromosome (Fig. S9). In particular, the intron span increases with respect to gene number were much smaller in ChrII and ChrV than in other chromosomes.

### Species description (continued)

#### Adult

Gonochoristic species. Middle to small sized species as the genus, ca. 1 mm in length. Tube-like stoma, ca. four times as wide as deep, with separated into three parts, cheilostom, gymnostom and stegostom from anterior as typical of the rhabditid nematodes. Cheilostom short tube-like occupying ca. 1/4 of total stoma. Gymnostom simple tube-like, as long as cheilostom. Stegostom covered by pharyngeal sleeve and separable from gymnostom, separated into four subsections, pro-, meso-, meta- and telostegostom. Metastegostom forming three well-sclerotized flap-like teeth, one on each dorsal, right and left subventral sector, the outer part weakly sclerotized to form a ring surrounding the posterior part of metastegostom. Excretory pore located around the level of or slightly posterior to basal bulb. Deirid can be observed laterally on lateral field at the almost same level with secretory-excretory pore.

#### Male

Gonad single-armed on the right subventral of intestine, composed by testis (anterior 2/3) and vas deferens (posterior 1/3). Testis anteriorly reflexed rightwardly. Tail enveloped by a closed bursa, supported by nine pairs of genital papillae (bursal rays). Anterior cloacal lip with a rounded and sclerotized appendage and bulge-like appendage present between rounded appendage and cloacal opening; a small sensilla-like papilla on the bulge-like appendage. Posterior cloacal lip with tongue-like appendage with two cloacal sensilla. Spicules paired, separate, long and moderately slender with evenly slightly ventrally curved blade (calomus-lamina complex) and simply pointed tip. Gubernaculum slender, ventrally arcuate with small squared appendage at the distal end in lateral view. Bursa anteriorly closed with serrated edge; terminal notch present but unclear. The nine pairs of bursal rays with an arranged (2/1 + 1 + 2 + 3). The r3 and r9 almost reach the edge of bursal velum; the r1, r5 and r7 opening dorsally forming papilliform tip; and r2, r6, r4, r8 opening ventrally forming papilliform tip. The r6 with expanded root, and the ray forming bowling pin-shape. Phasmids sensilla-like, ventrally directed between r8 and r9.

#### Female

Gonadal system didelphic, amphidelphic. Each gonadal system arranged as ovary, oviduct, spermatheca, spermathecal-uterus junction tissue, uterus and vulva/vagina from distal. Tail elongated conical with pointed tip. Phasmid forming small pore located laterally at ca. 1/3 of total tail length from anus, or ca. 1.9 anal body diam. Posterior to anus.

## Discussion

We here reported a new *Caenorhabditis* species named *C. niphades* n. sp., which is tightly associated with weevil (*N. variegatus)*. Although > 50 species have been described in the genus, the chromosome-level assembly provided for this species can be a useful resource to understand genome evolution in the genus. Intriguingly, we found *C. niphades* n. sp. genome size to be the smallest among all *Caenorhabditis* genomes reported so far; approximately 42 Mb smaller than *C. elegans’*.

The phylogenetic relationship among species in the genus *Caenorhabditis* have been well characterized previously [5–7, 20]; the genus has been mainly divided into three supergroups (Elegans, Drosophilae, and basal), where the Elegans supergroup has been further divided into the Elegans and Japonica groups. However, as described in Stevens et al., the relationship among *C. afra*, *C. sulstoni,* and *C. japonica* could not be well resolved by SNV-based phylogenetic analyses [7]. Our genome-based phylogenetic analysis showed an almost consistent result with the Stevens’ phylogeny; *C. niphades* n. sp. was located in the not-well-resolved cluster. However, *C. niphades* n. sp. appears to belong to the Japonica group, being the closest relative of *C. japonica.* Two other small-genome species in the Japonica group (*C. sulstoni* and *C. afra*) were positioned at the sister clade of the *C. niphades* n. sp. in our phylogenetic trees. Altogether, the genome size difference between *C. niphades* (59.0 Mb) and *C. japonica* (~ 166.3 Mb) can serve as a platform to investigate genome size evolution. Although the publicly available version of *C. japonica* genome assembly [7] requires an adequate reduction of haplotypes, our newly-generated assembly seems to represent the more realistic haploid genome size (141.2 Mb).

In some species, genome reduction is accompanied with loss of unnecessary genes and/or gene families. For example, in symbiotic bacteria genome reduction is part of a process that often includes preferential loss of genes in metabolic pathways and a decreased GC content [21]. Despite having the smallest genome in the Elegans supergroup, *C. niphades* n. sp. retains the core *Caenorhabditis* genes, including metabolic pathways- and embryonic development-related genes, indicating a compact genome in the ancestral Japonica group and that genome size expansion occurred during the evolution of some lineages by TE expansions and/or gene family expansion. Intriguingly, we found that the GC content in the Japonica group showed a negative correlation with genome size, the opposite to the phenomena observed in symbiotic bacteria [21], and a trend not found for the Elegans or basal groups. The GC content of *C. bovis*, which belongs to the basal group and has a small genome (62.7 Mb), is similar to *C. elegans*, implying variable genome evolution even within the genus.

We found well conserved synteny between *C. niphades* n. sp. and *C. elegans,* as reported in other *Caenorhabditis* species. Our chromosome-level assembly enabled us to compare each chromosome with its *C. elegans* counterpart. We found a small size and gene number of all *C. niphades* n. sp.’s chromosomes in autosomes, but the sex chromosome size difference was small and the gene number even bigger in *C. niphades* n. sp., indicating that sex chromosomes underwent a distinct evolutionary path from autosomes. As genome annotation of the new genome assembly of *C. japonica,* which is more closely related and has a bigger genome size than *C. elegans*, is under development in our laboratory, we did not perform a detailed comparison herein. However, a detailed comparison between these two species would provide a fascinating insight into chromosome evolution, including gene family evolution, transposon dynamics, and regulatory processes.

## Materials and methods

### Nematode isolation and culturing

Adults of *Niphades variegatus* (Roelofs) (Coleoptera: Curculionidae) were collected from the dead log of Masson’s pine, *Pinus massoniana* Lambert at Tama Forest Science Garden of Forestry and Forest Products Research Institute (GPS code: 35°38′59″ N, 139°16′29″ E, 201 m a.s.l) on 23 May, 2014. Collected weevils were brought back to laboratory alive, and stored at room temperature until dissection. Then, the weevils were dissected individually on 2.0% agar plate and observed under dissecting microscope to examine their nematode associations. When nematodes were found, their developmental stages and feeding habitats, assumed from the morphology, were noted and transferred to culture plate (*Botrytis cinerea* Pers. growing on 2.0% malt extract agar for fungal feeders, or *Escherichia coli* OP50 growing on NGM agar plate for bacteria feeders). The dissected weevil bodies on the agar plates were kept at room temperature and observed occasionally for 2 weeks to examine nematodes’ propagation. Propagated nematodes were transferred to a new culture plate. Those nematode culture plates were incubated at room temperature (23–25 °C) to establish laboratory cultures.

The weevils collected from dead logs of *Abies sachalinensis* F. Schmidt (42°59′38″ N, 141°23′33″ E, 170 m a.s.l) and *Pinus densiflora* Sieb. et Zucc. (42°59′44″ N, 141°20′55″ E, 78 m a.s.l) at Sapporo, Hokkaido on 13, May, 2016; *A. sachalinensis* at Esashi, Hokkaido (44°56′55″ N, 142°34′28″ E, 15 m a.s.l) on 15, May, 2016; *P. densiflora* at Sugadaira, Nagano (36°31′31″ N, 138°20′52″ E, 1331 m a.s.l) on 8 June, 2017; and *P. densiflora* at Kyoto, Kyoto (34°56′28″ N, 135°46′24″ E, 61 m a.s.l) on 15, May, 2018 were also examined with same methodology. In addition, other insects obtained from same logs, e.g., bark beetles, longhorn beetles and weevils, were examined for nematodes with same methodology.

Gravid females from a one-week-old culture were individually transferred to NGM plates seeded with *E. coli* OP50–1, and incubated at 25 °C. This procedure was repeated 5 times for the wild-type strain NKZ391. The last generation was subcultured and maintained as the isogenic line of *C. niphades* n. sp. with a culture code NKZ392.

### Morphological observation and morphometric analysis

Morphological characters were examined using fixed and mounted materials and live cultures. Adult nematodes from 1-week-old cultures were collected with pouring distilled water to culture media, heat-killed and fixed in TAF fixative (2:7:91 triethanolamine:formalin:distilled water solution) for a few days, processed in glycerine following the modified Seinhorst method [22], and mounted in glycerine according to the methods of Maeseneer and d’Herde [23]. These materials were designated as the type specimens, and were used for morphometric analysis. Live materials from separate 1-week-old cultures were used for morphological observations, and light photomicrography was performed using a microscope (Eclipse 80i; Nikon, Tokyo, Japan) with differential interference contrast optics, a drawing tube, and a microscopic digital camera (MC170 HD; Leica, Wetzlar, Germany) as described previously [24]. Drawings and micrographs were edited using the Photoshop Elements 2020 software (Adobe Inc., San Jose, CA, USA) to construct figures.

### Genome sequencing and assembly

Mixed-stage *C. niphades* n. sp. isogenic line (NKZ392) were washed out from the NGM culture plates and cleaned by discontinuous sucrose gradient centrifugation to remove culture debris [25]. The worms were incubated in a lysis solution ((Qiagen buffer G2 with 400 μg/ml proteinase K, 50 mM dithiothreitol, 0.5 mg/ml RNaseA (Invitrogen)) at 55 °C for 4 h. High-molecular-weight genomic DNA was extracted by phenol-chloroform extraction and ethanol precipitation. A Nanopore library was prepared using 1 μg genomic DNA using a sequencing kit SQK-LSK109 (Oxford Nanopore Technologies) according to the manufacturer’s protocol. A Nanopore sequencing run was performed with MinION R9.4.1 flow cells to obtain 8.19 Gb of sequence data (3216 K reads; N50, 6.2 kb). The Nanopore reads were base called using the Guppy v4.0.15 basecaller (Oxford Nanopore Technologies) with the dna_r9.4.1_450bps_hac configuration. An Illumina paired-end sequencing library was prepared from 100 ng of DNA using the Nextera DNA sample preparation kit and sequenced on Miseq using MiSeq Reagent Kit v3 according to the manufacturer’s protocol (Illumina) to obtain a total of 4.58 Gbp of paired-end reads (301 bp × 2). A HiC library was constructed from ∼5000 fresh worms using an Arima-HiC+ kit (Arima Genomics Inc.) and a Collibri ES DNA library prep kit (Thermo Fisher Scientific) according to the manufacturers’ protocols and was sequenced using a MiSeq instrument with the MiSeq reagent kit v3 (101 cycles × 2), to obtain 3.6 million paired-end reads.

The Nanopore long reads were assembled using Flye v2.7.1 [26] and base-corrected by three rounds of Pilon v1.23 [27] with the Illumina paired-end reads. Hi-C data was used to further scaffold the contigs using Arima-HiC Mapping Pipeline (v02, Arima Genomics Inc.), 3D-DNA pipeline v180114 with parameters: -g 50 -e [28] and Juicebox v1.11.08 [29] for visualization of the Hi-C results (Fig. 2).

*C. japonica* new genome assembly (v2.0) was generated from PacBio RS II read using P6-C4 chemistry (PacBio). The initial assembly was obtained using NextDenovo v.2.4.0 (https://github.com/Nextomics/NextDenovo) and base-corrected by three rounds of Pilon v1.23 [27] with the Illumina paired-end reads. Then, Hi-C reads generated using an Arima-HiC+ kit (Arima Genomics Inc.) were used to obtain a chromosome level assembly as described above.

Completeness of the assemblies were assessed using BUSCO v5.1.2 [30] with nematoda_odb9 single copy universal conserved genes.

### Gene prediction

Hisat2 v2.1.0 was used to map RNA-seq reads to the assembly to generate corresponding bam file and the bam file was further processed to produce intron hints by bam2hints script (parameters: --intronsonly --remove_redundant --minintronlen = 20 --maxintronlen = 7000) implemented in Augustus. Using this hint file, the initial protein-coding gene models were predicted by Augustus with the parameters (−-allow_hinted_splicesites = atac –species = celegans). A selection of the gene models (~ 1000) was manually curated in Artemis tool (Release 18.0.3) and used to train Augustus species parameters. The final gene models were generated with the trained Augustus and the hint file.

### Phylogenetic analysis

Full length of small subunit ribosomal RNA (SSU rRNA) and D2-D3 regions of large subunit ribosomal RNA (LSU rRNA) were sequenced for preliminary phylogenetic analysis. These regions were as described in Dayi et al. [31]. The sequences were aligned with those from 35 *Caenorhabditis* species using MAFFT [32] and the base substitution model was determined using Modeltest ver. 3.7 [33] under the Akaike information criterion model selection criterion. Then, a Bayesian analysis was performed to infer the tree topology using MrBayes 3.2 [34]; four chains were run for 5 × 10^6^ generations. Markov chains were sampled at intervals of 100 generations [35]. Two independent runs were performed, and the remaining topologies were used to generate a 25% majority-rule consensus tree after confirming convergence of runs.

To generate genome-based phylogenetic trees, we obtained 313 single-copy gene sequences from *C. niphades* n. sp., *C. auriculariae*, *C. bovis* other 32 *Caenorhabditis* species which were used in Stevens et al. [7] using OrthoFinder (version 2.2.6) [36]. Amino acid sequences of the single copy genes were aligned using MAFFT (version 7.455) [32], poorly aligned regions were trimmed by GBlocks (version 0.91b) [37] and finally 308 alignments were remained. The alignments were concatenated into a supermatrix and used as input for maximum likelihood (ML) tree generation using RAxML (version 8.2.12) [38] with -m PROTGAMMAAUTO -N 500 and partition options. A Bayesian analysis was performed using MrBayes 3.2 [34] using the concatenated alignments using the WAG substitution model; four chains were run for 5 × 10^6^ generations. A supertree approach using ASTRAL-III (version 5.7.3; option: -q) with 308 gene trees of the single copy genes was also employed to infer phylogenetic relationship [39].

### Synteny analysis

The best reciprocal BLAST-hit approach [11] was used to infer syntenic relationship between *C. niphades* n. sp. and *C. elegans* and it was visualized by TBtools [40].

### Repetitive sequences detection

RepeatModeler2 [41] and transpsonPSI (http://transposonpsi.sourceforge.net/) were used to determine repetitive element repertoire in *C. niphades* n. sp. genome. A consensus non-redundant repeat library was obtained by merging the outputs of these tools using USEARCH (v7.0) (option: --id 0.8). RepeatMasker (version 4.1.0) (https://www.repeatmasker.org/) (default option) was then used to mask repetitive elements in the genome.

## Supplementary Information

**Additional file 1. **Supplementary information 1 (Species Description). **Figure S1** (Phylogenetic relationships of *C. niphades* n. sp. and the other 26 *Caenorhabditis* species). **Figure S2** (Adults of *Caenorhabditis niphades* n. sp.). **Figure S3** (Left lateral view of the anterior region of adult female of *Caenorhabditis niphades* n. sp.). **Figure S4** (Scanning electron micrographs of male *Caenorhabditis niphades* n. sp.). **Figure S5** (Female characters of *Caenorhabditis niphades* n. sp.). **Table S2** (Repetitive element comparison). **Figure S6** (Schematic overview of six big contigs of the *C. niphades* n. sp. genome). **Figure S7** (Hi-C contact map of the assembled chromosome-length scaffolds for *C. niphades* n. sp.). **Figure S8** (Species-specific genes and Orthologues between various *Caenorhabditis* species). **Figure S9** (Relationship between gene number and genomic feature size in *C. niphades* n. sp. and *C. elegans*).

## Data Availability

All sequence data from the genome projects have been deposited at DDBJ/ENA/GenBank under BioProject accession PRJEB53466; the genome assembly is available from https://www.ebi.ac.uk/ena/browser/view/CAMPFS010000001-CAMPFS010000010 and the raw sequence data from https://www.ebi.ac.uk/ena/browser/view/ERR10147410, ERR10147413, and ERR10147416. The new *C. japonica* genome assembly and other *C. niphades* files are available from *Caenorhabditis* genomes project server (http://caenorhabditis.org/). All other relevant data are available from the authors with a request to T.K. (taisei.kikuchi@edu.k.u-tokyo.ac.jp).
