# Supplementary material for: The compact genome of Caenorhabditis niphades n. sp., isolated from a wood-boring weevil, Niphades variegatus
Source: BMC Genomics. 2022 Nov 22;23:765. doi: 10.1186/s12864-022-09011-8 (PMC9682657; doi:10.1186/s12864-022-09011-8)
Supplement: Supplementary file 1 — Additional file 1. Supplementary information 1 (Species Description). Figure S1 (Phylogenetic relationships of C. niphades n. sp. and the other 26 Caenorhabditis species). Figure S2 (Adults of Caenorhabditis niphades n. sp.). Figure S3 (Left lateral view of the anterior region of adult female of Caenorhabditis niphades n. sp.). Figure S4 (Scanning electron micrographs of male Caenorhabditis niphades n. sp.). Figure S5 (Female characters of Caenorhabditis niphades n. sp.). Table S2 (Repetitive element comparison). Figure S6 (Schematic overview of six big contigs of the C. niphades n. sp. genome). Figure S7 (Hi-C contact map of the assembled chromosome-length scaffolds for C. niphades n. sp.). Figure S8 (Species-specific genes and Orthologues between various Caenorhabditis species). Figure S9 (Relationship between gene number and genomic feature size in C. niphades n. sp. and C. elegans). [file 12864_2022_9011_MOESM1_ESM.pdf]

Supplementary information 1 for

The compact genome of *Caenorhabditis niphades* n. sp., isolated from a wood-boring weevil, *Niphades variegatus*

Species description of *Caenorhabditis niphades* n. sp.

Taxonomic lineage

Phylum Nematoda Diesing, 1861

Class Chromadorea Inglis, 1983

Order Rhabditida Chitwood, 1933

Family Rhabditidae Örley, 1880

Genus *Caenorhabditis* Osche, 1952

Caenorhabditis niphades n. sp.

Description

Typological characters are provided as the drawings (Supplementary Figure 2) and photomicrographs (Supplementary Fig. 2-4), and the morphometric values are provided in Supplemental Table 1.

Supplementary Table 1. Morphometric values of *Caenorhabditis niphades* n. sp.

	Holotype male	Paratype males	Paratype females
n	-	9	10
L	780	809 ± 47 (759-897)	1070 ± 71 (894-1125)
a	18.6	18.5 ± 1.1 (16.8-20.4)	18.5 ± 1.8 (15.9-20.9)
b	5.2	5.5 ± 0.3 (5.1-6.2)	6.5 ± 0.2 (6.1-7.8)
c	15.5	18.3 ± 1.5 (15.5-20.7)	7.2 ± 0.5 (6.4-7.8)
c’	2.0	1.7 ± 0.1 (1.6-2.0)	5.5 ± 0.7 (4.4-6.6)
T[1)] or V	70.3	74.2 ± 4.2 (68.2-79.6)	52.0 ± 1.4 (48.6-53.3)
Maximum body diam.	42	44 ± 3.5 (40-50)	58 ± 6.3 (53-70)
Stomatal opening diam.	4.3	4.3 ± 0.3 (3.9-4.6)	4.9 ± 0.6 (4.3-5.7)
Stoma diam. at mid part	3.5	3.4 ± 0.2 (3.2-3.9)	3.9 ± 0.4 (3.5-4.6)
Stoma depth	14.9	14.3 ± 1.1 (11.7-15.6)	14.9 ± 0.6 (14.2-16.0)
Stoma depth/diam. ratio	4.2	4.2 ± 0.4 (3.7-4.9)	3.8 ± 0.4 (3.3-4.5)
Anterior pharynx length	70	70 ± 3.5 (63-76)	79 ± 4.0 (69-83)
Posterior pharynx length	64	63 ± 3.2 (56-67)	69 ± 4.4 (64-80)
Anterior/posterior pharynx	1.09	1.12 ± 0.1 (1.06-1.24)	1.14 ± 0.1 (1.0-1.24)

ratio			
Nerve ring from anterior end	112	107 ± 7.4 (94-117)	123 ± 5.5 (110-127)
Secretory-excretory pore from anterior end	140	139 ± 7.6 (130-154)	162 ± 7.1 (150-171)
Deirid from anterior end	143	140 ± 8.1 (130-154)	162 ± 6.4 (150-170)
Median bulb diam.	17.9	18.2 ± 1.2 (16.7-20.2)	22.1 ± 0.9 (20.2-23.2)
Basal bulb diam.	19.0	19.0 ± 0.9 (17.9-20.2)	23.5 ± 0.6 (22.6-24.4)
Cloacal or anal body diam.	26	27 ± 0.9 (25-28)	27 ± 1.9 (24-30)
Tail length	50	44 ± 2.9 (40-50)	149 ± 16 (121-173)
Gonad length[2)]	548	600 ± 45 (539-675)	-
Reflex part of testis	100	102 ± 20 (79-143)	

1) The ratio of gonad to whole body length where the gonad length includes reflex part of testis and *vas deferens*.
2) The gonad length includes reflex part of testis and *vas deferens*.
3) Curved length from vulval opening to the tip of ovary, i.e., including uterus, oviduct, spermathecal and ovary.
4) Ratio of anus-phasmid distance to anal body diam.
5) Relative position of phasmid to total tail length in %.

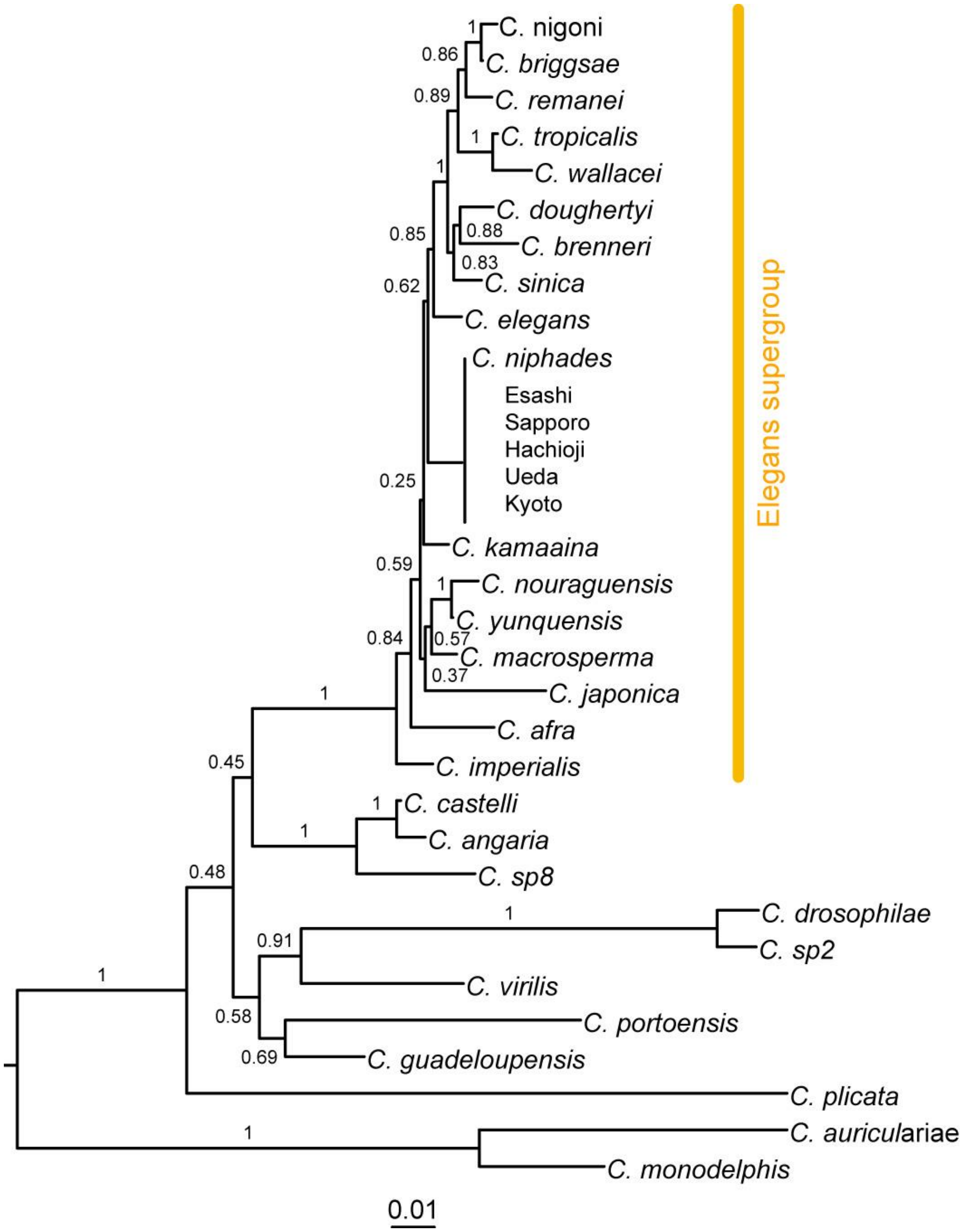

Figure S1. Phylogenetic relationships of *C. niphades* n. sp. and the other 26 *Caenorhabditis* species inferred by near-full-length 18S and D2/D3 region of 28S ribosomal RNA genes. Nucleotide sequences were aligned before concatenation, and maximum-likelihood trees were generated with RAxML v7.2.8 using the best-fitting empirical model with 1,000 bootstrap resampling replicates with the support percentage shown on the nodes. The scale bar shows nucleotide substitutions per site.

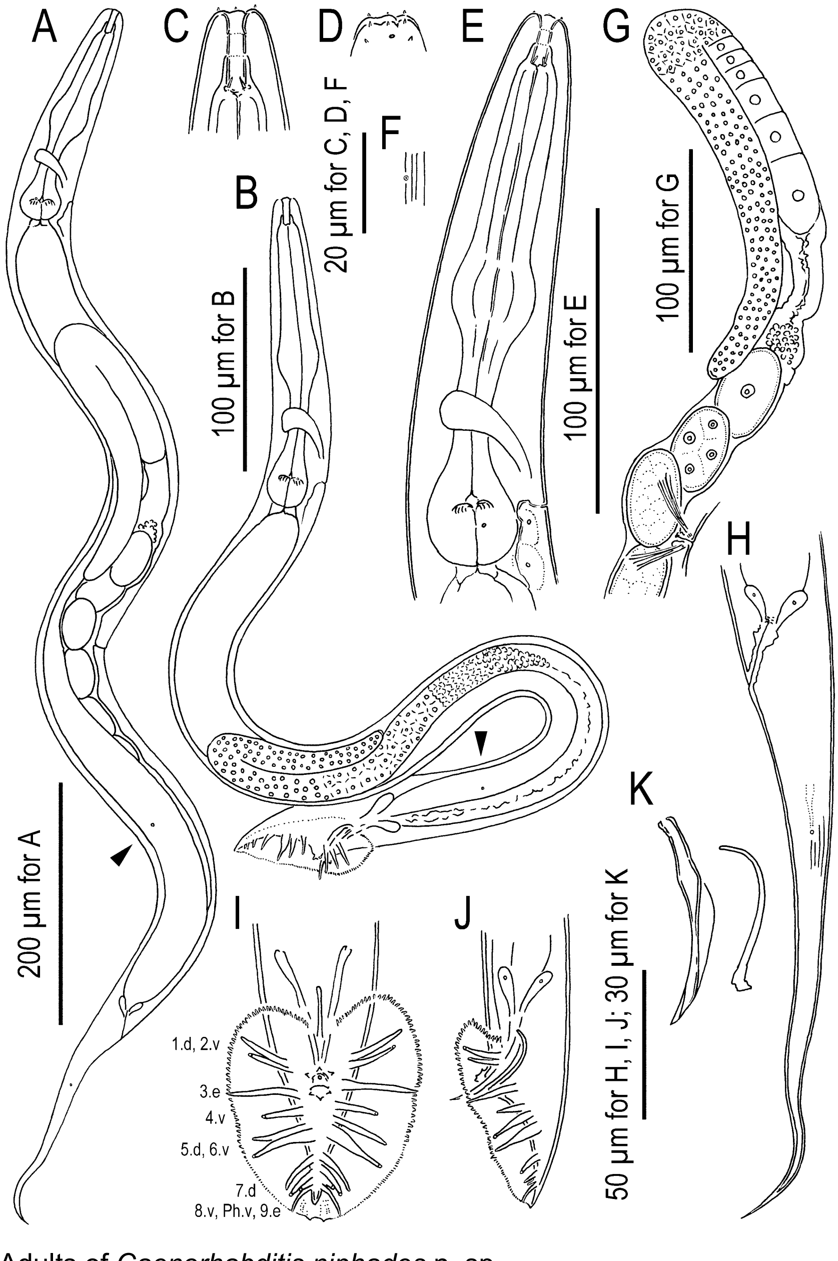

Figure S2. Adults of *Caenorhabditis niphades* n. sp.

A: Whole female body in right lateral view; B: Whole male body in right lateral view; C: Male stomatal region in left lateral view; D: Male stomatal opening region in left lateral view; E: Male stoma and pharynx region in right lateral view; F: Lateral field and deirid of male in right lateral view; G: Anterior female gonad in right lateral view; H: Female tail region in left lateral view; I: Male tail in ventral view; J: Male tail in left lateral view; K: Spicule and gubernaculum in left lateral view. Postdeirid position is indicated by arrowheads (A, B), and the direction of genital papillae (number) and phasmid (Ph) are indicated with a form 'number/Ph.direction' in I, where, 'd', 'v', and 'e' suggest the papillae or phasmid open to dorsal side, ventral side or reach to the edge, respectively.

Adult.

Gonochoristic species. Middle to small sized species as the genus, ca. 1 mm in length. Cuticle is moderate in thickness with fine transverse annulations and longitudinal striations. Lateral field with four lines. Deirids clearly observed laterally on the lateral field, at the level of or slightly behind the secretory-excretory pore. Postdeirid can be observed, and the position is described for each sex. Lip region not clearly offset, i.e., not clearly separated from the other parts of body, separated into six lip sectors. Each lip sector has a labial sensillum, and four cephalic sensilla, one on each dorsal and subventral sector in males. Amphidial apertures small, oval pore-like, visible laterally, slightly dorsally located as in other rhabditid and diplogastrid nematodes, at the level of the margin of cheilo and gymnostom. Tube-like stoma, ca. four times as wide as deep, separated into three parts, cheilostom, gymnostom and stegostom from anterior as typical of the rhabditid nematodes. Cheilostom short tube-like occupying ca. 1/4 of total stoma, posteriorly overlapping with the anterior end of gymnostom. Gymnostom simple tube-like, as long as cheilostom, i.e., occupying ca. 1/4 of total stoma, weakly separated into two subsections, but the separation is difficult to observe in microscopic observation, each subsection is hypothesized to be associated with an arcade synsitium. Stegostom covered by pharyngeal sleeve and separable from gymnostom, separated into four subsections, pro-, meso, meta- and telostegostom. Pro- and mesostegostom not clearly separated, fused to form a simple tube which is almost same as cheilo + gymnostom in length. Metastegostom forming three well-sclerotized flap-like teeth, one on each dorsal, right and left subventral sector, the outer part weakly sclerotized to form a ring surrounding the posterior part of metastegostom. Telostegostom without clear armature, forming small funnel connecting stoma and pharynx (procorpus). Pharynx separated into four sections, procorpus, metacorpus (median bulb), isthmus and basal bulb. Pro- and meta corpus forming muscular anterior pharynx and the other two sections form glandular posterior pharynx. Procorpus muscular tube occupying ca. 60% or a little more of corresponding body diam. Metacorpus forming muscular median bulb without clear valve or glottoid apparatus. Isthmus narrow, not muscular. Basal bulb well-developed with double haustrulum as the glottoid apparatus (grinder-like structure typical to rhabditid nematodes). Pharyngo-intestinal valve (cardia) prominent. Nerve ring around the middle of isthmus. Excretory pore located around the level of or slightly posterior to basal bulb, perpendicular to body surface and possessing tube-like excretory-secretory duct. A large cell visible on ventral side, at level of, or a little posterior to, cardia, assumed to be a secretory cell associated with secretory-excretory system.

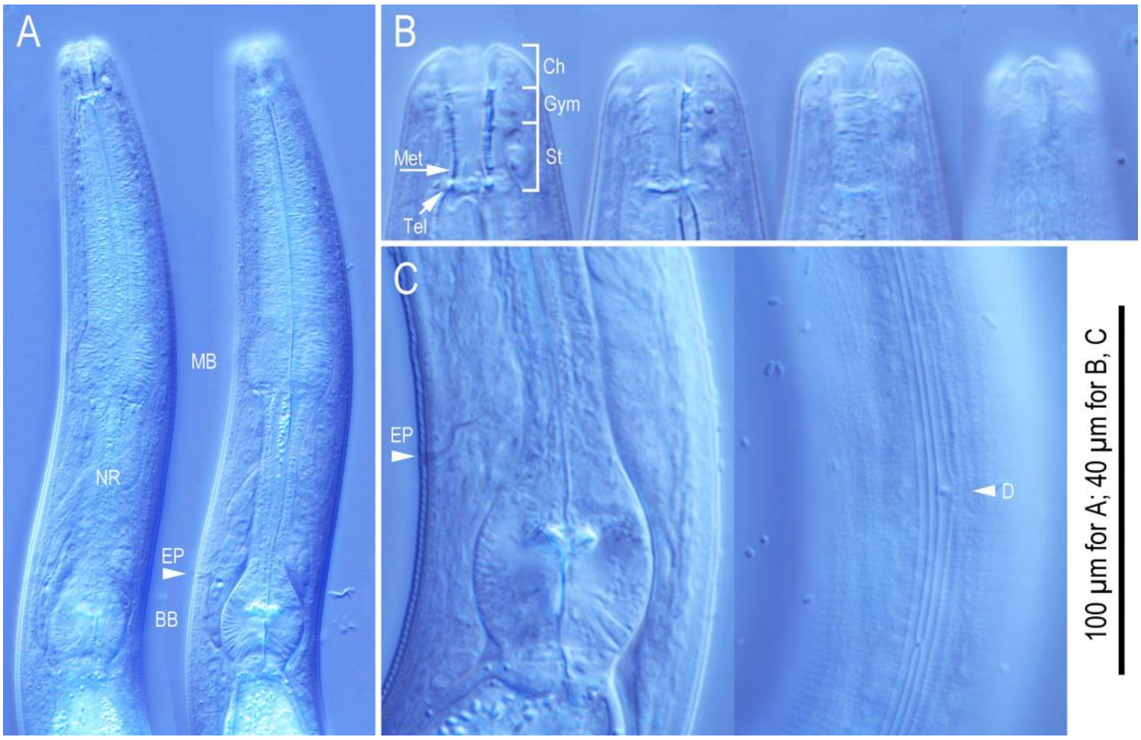

Figure S3. Left lateral view of the anterior region of adult female of *Caenorhabditis niphades* n. sp. A: Stoma and pharynx (MB: Median bulb; NR: Nerve ring; EP: Secretory-excretory pore; NN: Basam bulb); B. Stomatal region in four focal planes (Ch: Cheilostom; Gym: Gymnostom; St: Stegostom; Met: Metastegostomatal flaps; Tel: Telostegostomatal ring); C: Basal bulb region in two different focal planes showing the relative position of the basal bulb, secretory-excretory pore and deirid (EP: Secretory-excretory pore; D: Deirid on the lateral field).

Male.

Postdeirid slightly shifted dorsally around the level of middle part of *vas deferens*. Gonad single-armed on the right subventral of intestine, composed by testis (anterior 2/3) and *vas deferens* (posterior 1/3). Testis anteriorly reflexed rightwardly. Spermatocytes arranged in three to four rows in the reflexed part; well-developed spermatocytes in the two to three rows in the middle part; mature spermatids tightly packed in the rest of testis. *Vas deferens* composed of large cells, fused with the intestine (rectum) in its posterior end (at the level of spicule) to form a narrow cloacal tube. Tail enveloped by a closed bursa, supported by nine pairs of genital papillae (bursal rays). Anterior cloacal lip with a trapezoid-shaped appendage on which a small sensilla-like papilla present. Posterior cloacal lip with tongue-like appendage with two cloacal sensilla. Spicules paired, separate, long and moderately slender

with evenly slightly ventrally curved blade (calomus-lamina complex) and simply pointed tip. Gubernaculum slender, ventrally arcuate with small squared appendage at the distal end in lateral view; forming spindle shape with outwardly pointed appendage in ventral view. Bursa heart-shaped in ventral view, anteriorly closed with serrated edge; serratae obvious in anterior half and vague in posterior half; terminal notch present but unclear. The nine pairs of genital papillae or bursal rays supporting the bursal velum with an arranged (2/1+1+2+3), i.e., first and second rays (r1 and r2) anterior to cloacal opening, close (stuck) to each other, third ray adcloacal, forth ray slightly posterior to r3, fifth and sixth rays close (stuck) to each other and slightly posterior to r4, seventh to ninth rays (r7-9) grouped at the middle between r6 and tail tip. The r3 and r9 almost reach the edge of velum, others open dorsally or ventrally in the velum. The r1, r5 and r7 opening dorsally forming papilla-form tip, r2, r6, r4, r8 opening ventrally forming papilla-form tip. The r6 with expanded root, and the ray forming bowling pin-shape. Phasmids sensilla-like, ventrally directed between r8 and r9.

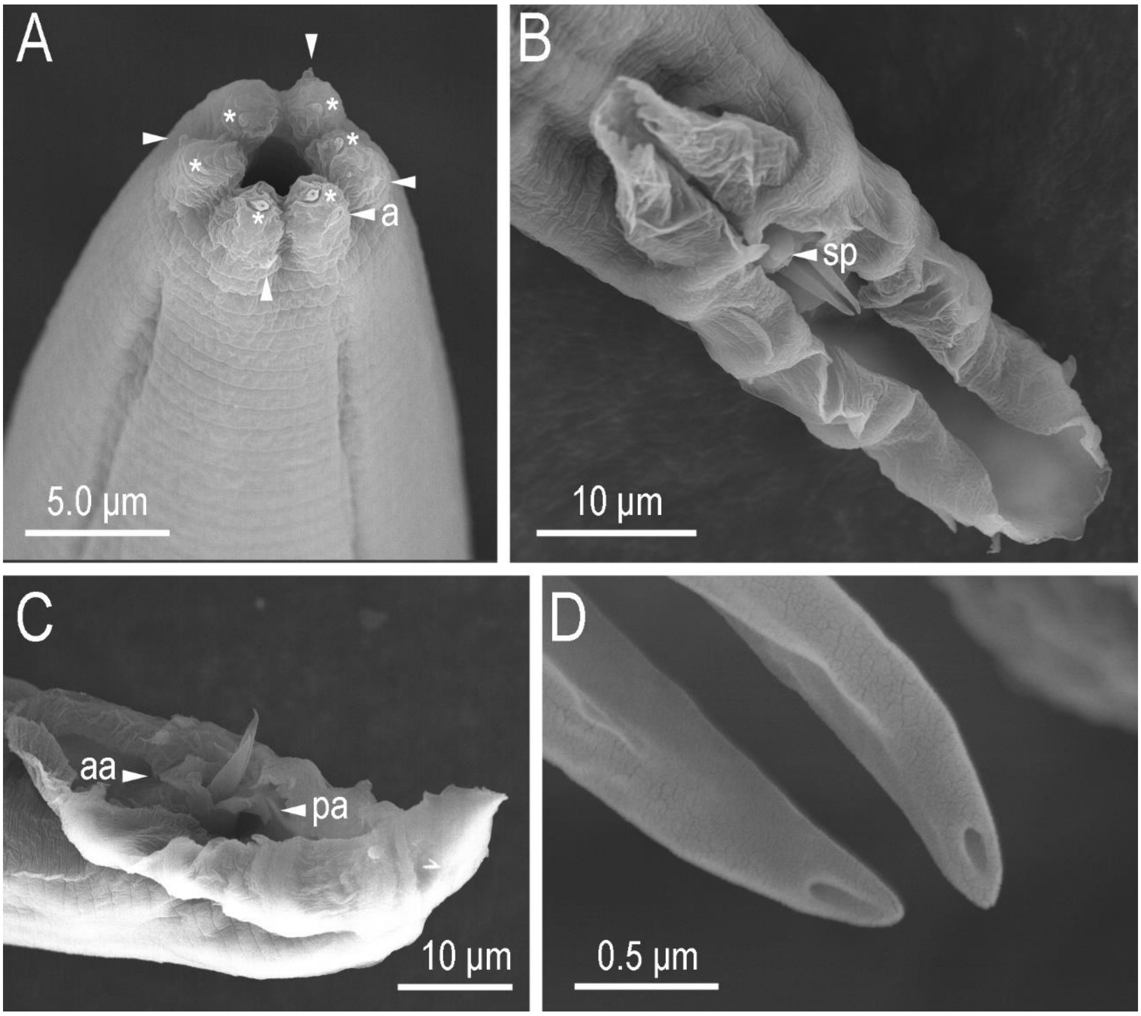

Figure S4. Scanning electron micrographs of male *Caenorhabditis niphades* n. sp. A: Left

latero-dorsal view of the stomatal region (a: amphid; arrowhead without character: cephalic sensilla; asterisk: labial sensilla); B, C: Ventral view of the tail region (sp: sensillum-like papilla; aa: anterior cloacal appendage; pa: posterior cloacal appendage); D: Spicule tip.

Female.

Body straight or slightly ventrally arcuate when killed by heat. Gonadal system didelphic, amphidelphic. Anterior and posterior gonadal system on the right and left of intestine, respectively, and basically symmetric with each other, thus anterior gonad is described from distal part to vulva/vagina. The gonadal system arranged as ovary, oviduct, spermatheca, spermathecal-uterus junction tissue, uterus and vulva/vagina from distal (anterior). Distal part of ovary reflexed dorsally; oocytes arranged in multiple (2-5) rows in the reflexed part, and well-developed oocytes arranged in single row near oviduct. Oviduct short composed with small and rounded cells connecting ovary and spermatheca. Spermatheca composed of large and squared cells forming roundish rectangular-shaped sac. Spermatheca-uterus junction not clearly observed because the part is often occupied by eggs; but in some individuals, a band of long cells with a band of thread-like appearance is surrounded by small and rounded cells. Uterus well-developed often containing some developing eggs. Vagina perpendicular to body surface constricted by sphincter muscle at the uterus-vagina junction. Vulva horizontal slit with vulval lips slightly protruding. Tail elongated conical with pointed tip. Anus and rectum clearly visible; the intestine-rectum junction constricted with sphincter muscle and surrounded by three (two subventral and one dorsal) rectal glands. Anal opening dome-shaped slit in ventral view. Phasmid forming small pore located laterally at ca. 1/3 of total tail length from anus, or ca. 1.9 anal body diam. posterior to anus.

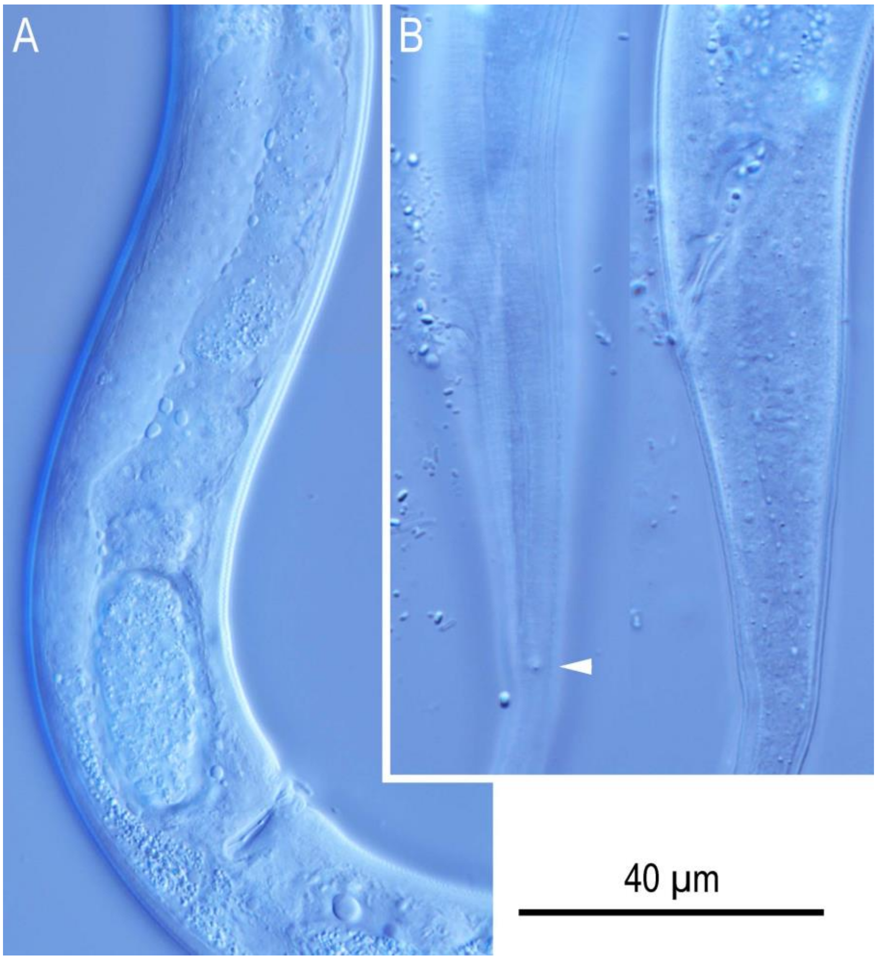

Figure S5. Female characters of *Caenorhabditis niphades* n. sp.

A: Anterior gonad in right lateral view; B: Anal region in left sublateral view in two different focal planes. The phasmid is indicated by an arrowhead (B).

Etymology

The species epithet is derived from the carrier insect, *Niphades variegatus* from that the nematode was isolated.

Materials examined

Holotype male, four paratype males, and five paratype females were deposited in the USDA Nematode Collection, Beltsville, MD, USA with the accession numbers T-793t (holotype male), T-7825p-7828p (four paratype males) and T-7829p-7833p (five paratype females), and five paratype males and five paratype females are deposited in the Forest Pathology Laboratory Collection of Forestry and Forest Products Research Institute (FFPRI), Tsukuba, Japan with accession numbers, *Caenorhabditis niphades* MP01-05, FP01-05. Several more unmounted specimens are available upon request from FFPRI (N. Kanzaki). In addition to

type materials, temporal water mount materials from cultures were examined for morphological observations. The type strain NKZ391 is available on request from Miyazaki University (T. Kikuchi).

Type and other localities

The type specimens of *C. niphades* n. sp. were collected from the cultured materials of NK391. The culture was established from population obtained from adult weevils, *Niphades variegatus* (Roelofs) collected from the dead log of Masson's pine, *Pinus massoniana* Lambert at Tama Forest Science Garden of Forestry and Forest Products Research Institute, Hachioji, Tokyo (GPS code: 35º38'59" N, 139º16'29" E, 201 m a.s.l) on 23 May, 2014. In addition to type locality, the species has been found from *N. variegatus* collected from dead logs of *Abies sachalinensis* F.Schmidt (42º59'38" N, 141º23'33" E, 170m a.s.l) and *P. densiflora* Sieb. et Zucc. (42º59'44" N, 141º20'55" E, 78 m a.s.l) at Sapporo, Hokkaido on 13, May, 2016; *A. sachalinensis* at Esashi, Hokkaido (44º56'55" N, 142º34'28" E, 15 m a.s.l) on 15, May, 2016; *P. densiflora* at Ueda, Nagano (36º31'31" N, 138º20'52" E, 1331 m a.s.l) on 8 June, 2017; and *P. densiflora* at Kyoto, Kyoto (34º56'28" N, 135º46'24" E, 61 m a.s.l) on 15, May, 2018.

Diagnosis and relationship

Based upon the arrangement of bursal rays and bursal velum shape, *C. niphades* n. sp. clearly belongs to Elegans supergroup (Sudhaus & Kiontke, 1996; Sudhaus, 2011; Kiontke et al., 2011; Felix et al., 2014; Huang et al., 2014). The members of the group are typologically close to, or almost identical to each other, except for *C. inopinata* (Kiontke et al., 2011; Kanzaki et al., 2018). In addition, the detailed morphological characters of the Elegans supergroup species, except for some key characters, have not been described, so far (Kiontke et al., 2011; Félix et al., 2014; Huang et al., 2014). Thus, it is difficult to distinguish *C. niphades* n. sp. from other Elegans supergroup species by the typological characters. However, *C. niphades* n. sp. has relatively wide stoma, i.e., width-length ratios are ca. 4.2 and 3.8 for males and females, respectively, and its composition, i.e., the ratio of chailo, gymno and stegostom is 1 : 1 : 2, may be characteristic to the species. Practically, the new species can be characterized only by its molecular phylogenetic status, i.e., *C. niphades* n. sp. is basal of the Elegans supergroup, and can be clearly distinguished from all other sequenced species by its phylogenetic status.

Remark on biological characters

Caenorhabditis niphades n. sp. has been isolated from *N. variegatus* from five different

localities, although two localities in Sapporo are close to each other. In addition, the nematode was not isolated from other coleopteran insects (bark and longhorn beetles and other weevil species) sharing the same log with *N. variegatus*. Therefore, the species is considered to have close and specific phoretic relationship with *N. variegatus*. The organ of the weevil harboring *C. niphades* n. sp. was not clarified, i.e., only propagated nematodes were recognized around the dissected body of the weevils on agar plate. That is partially because of *Bursaphelenchus niphades* Tanaka, Tanaka, Akiba, Aikawa, Maehara, Takeuchi & Kanzaki, i.e., *N. variegatus* often harbors large number of *B. niphades* dauers in its tracheal system (Tanaka et al., 2014), and these dauers mask the *C. niphades* n. sp. dauers during the dissection. More careful dissection survey is necessary to figure out the carrier utilization of *C. niphades* n. sp.

Remarks on typological characters In addition to the common characters of Elegans supergroup, *C. niphades* n. sp. has a characteristic wide stoma. This character is shared with several other species belonging to other species groups, e.g., *C. angaria* Sudhaus, Kiontke & Giblin-Davis, 2011, *C. auriculariae* Tsuda & Futai, 1999 and *C. parvicauda* Stevens, Félix, Beltran, Braendle, Caurcel, Fausett, Fitch, Frézal, Gosse, Kaur, Kiontke, Newton, Noble, Richaud, Rockman, Sudhaus & Blaxter, 2019 (Tsuda & Futai, 1999; Sudhaus et al., 2011; Stevens et al., 2019; Deyi et al., 2021). These species have been isolated from sugarcane and palm weevils (*C. angaria*), mushroom (*C. auriculariae*) and rotten plants (*C. parvicauda*) (Tsuda & Futai, 1999; Sudhaus et al., 2011; Stevens et al., 2019; Deyi et al., 2021), and their isolation sources (habitats) are not clearly different from other *Caenorhabditis* spp. (Kiontke et al., 2011; Stevens et al., 2019). Therefore, although the structure could be an adaptation to their habitat and feeding preferences, usefulness for the animals, i.e., functional advantage in their habitat, is not clear at current status.

References

Félix, M.A., Braendle, C. & Cutter, A.D. (2014). A streamlined system for species diagnosis in *Caenorhabditis* (Nematoda: Rhabditidae) with name designations for 15 distinct biological species. PLoS One 9, e94723. DOI:10.1371/journal.pone.0094723.

Huang, R. E., Ren, X., Qiu, Y. & Zhao, Z. (2014). Description of *Caenorhabditis sinica* sp. n. (Nematoda: Rhabditidae), a nematode species used in comparative biology for *C. elegans*. PLoS One 9, e110957. DOI:10.1371/journal.pone.0110957.

Kiontke, K.C. Félix, M.-A., Ailion, M., Rockman, M.V., Braendle, C., Pénigault, J.-B. & Fitch,

D.H.A. (2011). A phylogeny and molecular barcodes for *Caenorhabditis*, with numerous new species from rotting fruits. BMC Evolutionary Biology 11, 339. DOI:10.1186/1471-2148-11-339.

Sudhaus, W. (2011). Phylogenetic systematisation and catalogue of paraphyletic "Rhabditidae" (Secernentea, Nematoda). Journal of nematode morphology and systematics 14, 113-178.

Sudhaus, W. & Kiontke, K. (1996). Phylogeny of *Rhabditis* subgenus *Caenorhabditis* (Rhabditidae, Nematoda). Journal of Zoological Systematics and Evolutionary Research 34, 217-233. DOI: 10.1111/j.1439-0469.1996.tb00827.x

Tanaka, S.E., Tanaka, R., Akiba, M., Aikawa, T., Maehara, N., Takeuchi, Y. & Kanzaki, N. (2014). *Bursaphelenchus niphades* n. sp. (Tylenchina: Aphelenchoididae) amensally associated with *Niphades variegatus* (Roelofs) (Coleoptera: Curculionidae). Nematology 16, 259-281. DOI: 10.1163/15685411-00002763

Table S2. Repetitive element comparison. Repetitive element contents in *C. niphades* n. sp., *C. elegans*, *C. japonica*, *C. bovis*, *C. afra* and *C. sulstoni* were detected by combination of RepeatModeler2 (v 2.0.1) and transposonPSI.

	***C. niphades* n. sp.**		***C. elegans***		***C. japonica***		***C. bovis***		***C. afra***		***C. sulstoni***	
	**num element**	**% in bp**	**num element**	**% in bp**	**num element**	**% in bp**	**num element**	**% in bp**	**num element**	**% in bp**	**num element**	**% in bp**
SINEs	126	0.04	190	0.03	842	0.07	79	0.02	227	0.06	557	0.13
LINEs	2155	0.70	14556	2.23	66781	6.37	986	0.68	2418	0.77	2368	0.75
LTR element	4575	1.82	14959	2.90	118007	11.22	3714	1.65	5065	1.98	5142	2.01
DNA element	9438	2.45	58525	9.68	121897	10.75	7016	3.05	13511	2.86	13317	2.61
Rolling-circles	331	0.09	3004	0.61	3606	0.37	247	0.07	1781	0.48	856	0.30
Unclassified	17230	6.25	11684	2.54	187813	16.63	19371	4.64	13485	4.51	15160	4.77
Total interspersed repeats		11.27		17.38		44.04		10.03		10.18		10.26
Small RNA	567	0.24	540	0.08	1540	0.32	307	0.07	718	0.14	627	0.13
Satellites	132	0.04	358	0.06	2952	0.60	88	0.02	111	0.04	254	0.10
Simple repeat	18348	1.15	20587	1.02	34767	0.85	17252	1.03	28794	1.63	28287	1.70
Low complexity	3311	0.27	5816	0.27	9377	0.26	3311	0.24	6670	0.49	6114	0.47
Total		13.06		19.41		46.45		11.46		12.95		12.96

Table S3. Chromosome size, gene number, and GPCR gene number on each chromosome of *C. niphades* and *C. elegans*.

	**ChrI**		**ChrII**		**ChrIII**		**ChrIV**		**ChrV**		**ChrX**	
	C. elegans	*C. niphades*	*C. elegans*	*C. niphades*	*C. elegans*	*C. niphades*	*C. elegans*	*C. niphades*	*C. elegans*	*C. niphades*	*C. elegans*	*C. niphades*
**Chromosome size (bp)**	15M	9M	15M	9M	14M	9M	17M	9M	21M	10M	18M	13M
**gene number**	2911	2690	3514	2929	2689	2592	3311	2728	4953	3194	2769	2775
**GPCR gene number**	34	17	171	61	36	17	153	55	899	225	36	7

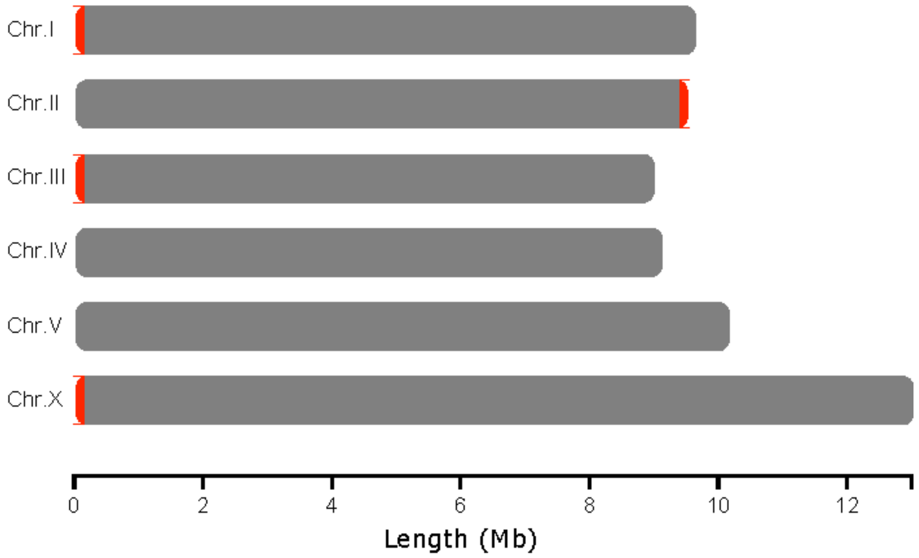

Figure S6. Schematic overview of six big contigs of the *C. niphades* n. sp. genome. Red color indicates positions where telomeric repeats (>20 TTAGGC unit) were detected.

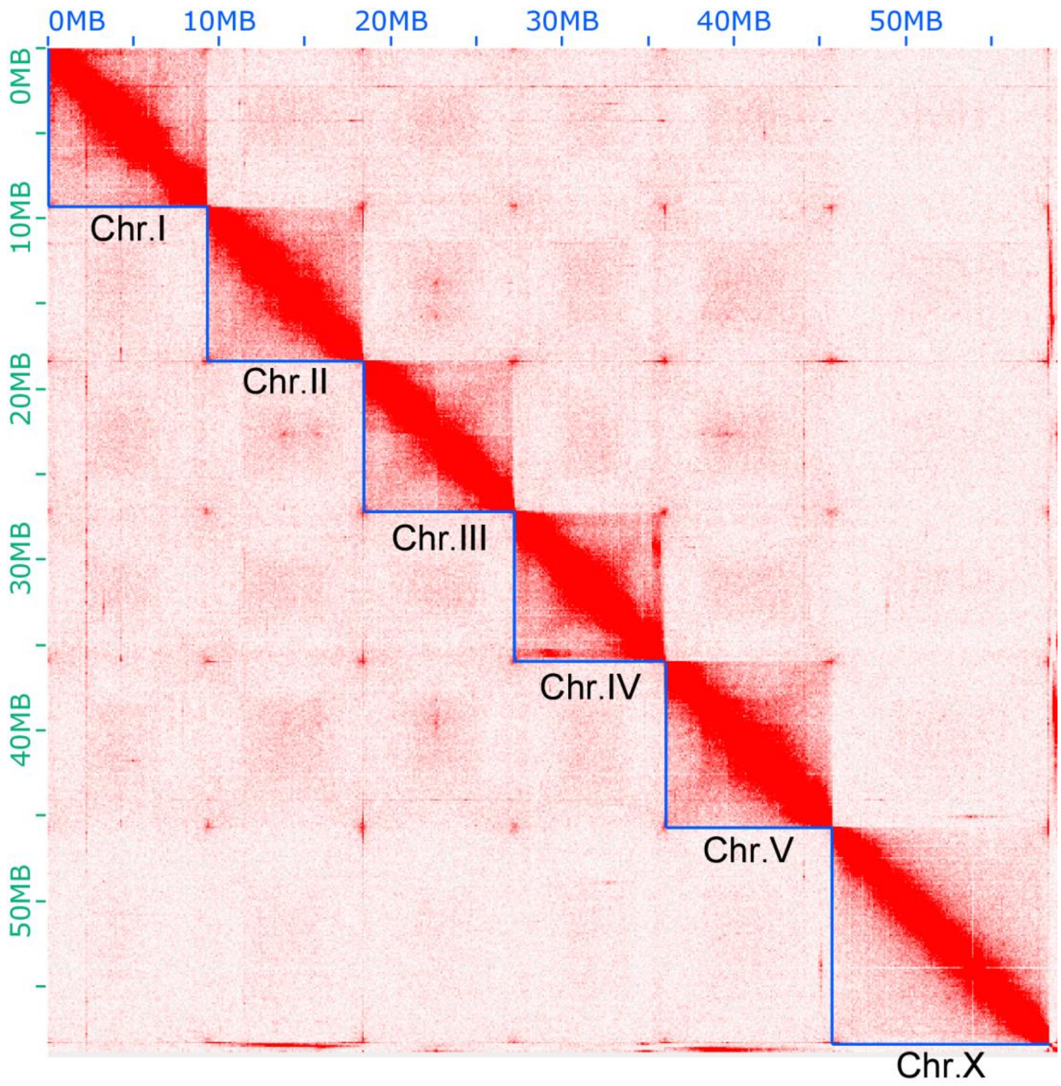

Figure S7. Hi-C contact map of the assembled chromosome-length scaffolds for *C. niphades* n. sp. The six scaffolds are indicated by blue lines, corresponding to the six chromosomes in *C. niphades* n. sp. genome.

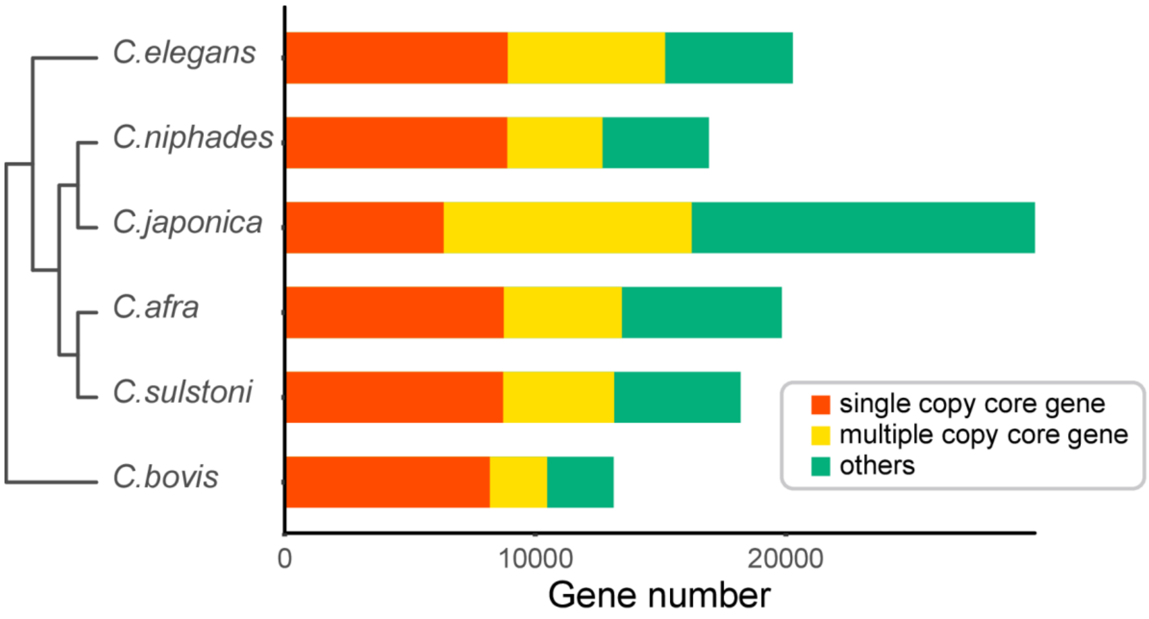

Figure S8. Species-specific genes and Orthologues between various *Caenorhabditis* species. Core genes were defined as those present in the core orthogroup (identified by Othofinder version 2.2.6), in which orthologues of *C. elegans*, *C. inopinata*, *C. nigoni*, and *C. briggsae* must be detected.

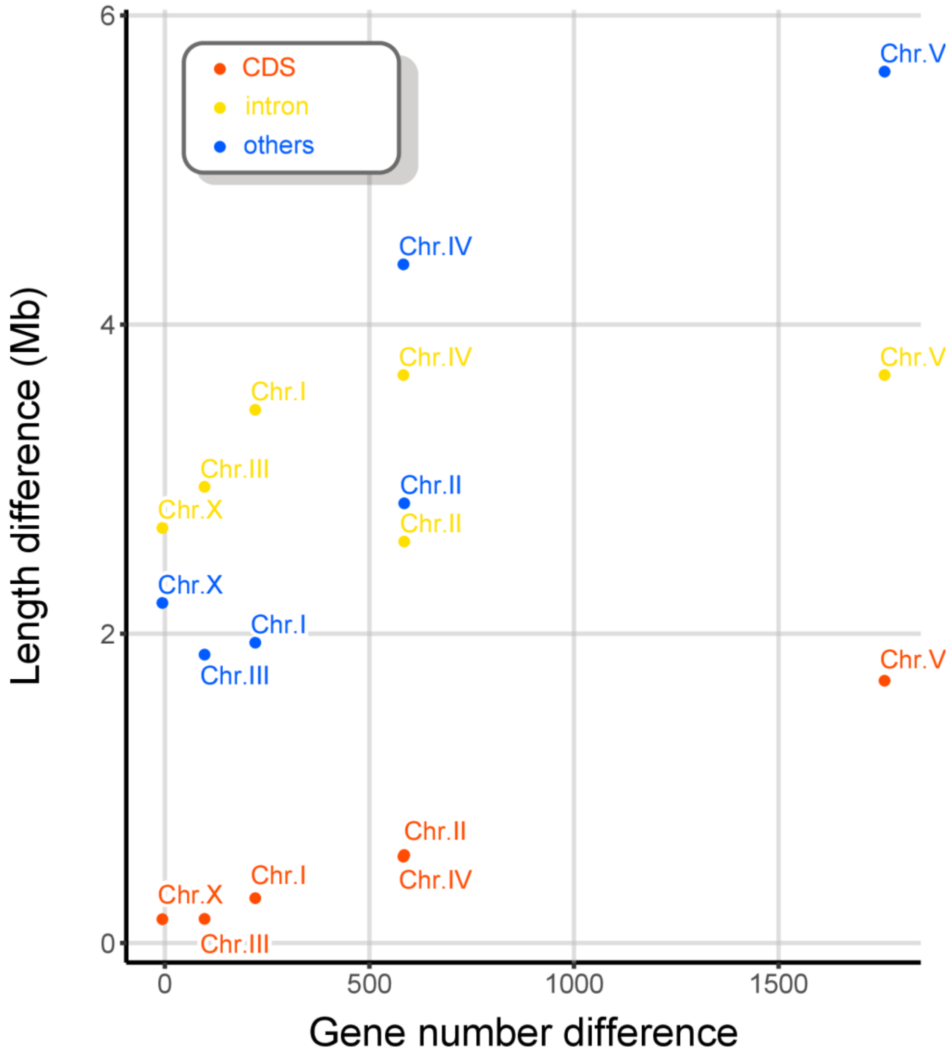

Figure S9. Relationship between gene number and genomic feature size in *C. niphades* n. sp. and *C. elegans*.
